# A standardized workflow for long-term longitudinal actigraphy data processing using one year of continuous actigraphy from the CAN-BIND Wellness Monitoring Study

**DOI:** 10.1038/s41598-023-42138-6

**Published:** 2023-09-15

**Authors:** Anastasiya Slyepchenko, Rudolf Uher, Keith Ho, Stefanie Hassel, Craig Matthews, Patricia K. Lukus, Alexander R. Daros, Anna Minarik, Franca Placenza, Qingqin S. Li, Susan Rotzinger, Sagar V. Parikh, Jane A. Foster, Gustavo Turecki, Daniel J. Müller, Valerie H. Taylor, Lena C. Quilty, Roumen Milev, Claudio N. Soares, Sidney H. Kennedy, Raymond W. Lam, Benicio N. Frey

**Affiliations:** 1 Department of Psychiatry and Behavioural Neurosciences, McMaster University, 100 West 5th Street, Suite C124, Hamilton, ON L8N 3K7 Canada; 2 Department of Psychiatry, Dalhousie University, Halifax, NS Canada; 3 Centre for Depression and Suicide Studies, St. Michael’s Hospital, Toronto, ON Canada; 4 Department of Psychiatry, Cumming School of Medicine, and Hotchkiss Brain Institute, University of Calgary, Calgary, AB Canada; 5 Mood Disorders Program, St. Joseph’s Healthcare Hamilton, Hamilton, ON Canada; 6 Campbell Family Mental Health Research Institute, Centre for Addiction and Mental Health, Toronto, ON Canada; 7 University Health Network, University of Toronto, Toronto, ON Canada; 8 Neuroscience, Janssen Research & Development, LLC, Titusville, NJ 08560 USA; 9 Department of Psychiatry, University of Michigan, Ann Arbor, USA; 10 Center for Depression Research and Clinical Care, UT Southwestern Medical Center, Dallas, TX USA; 11 Douglas Institute, Department of Psychiatry, McGill University, Montreal, QC Canada; 12 Department of Psychiatry, Queen’s University and Providence Care Hospital, Kingston, ON Canada; 13 Department of Psychiatry, University of Toronto, Toronto, ON Canada; 14 Department of Psychiatry, University of British Columbia, Vancouver, BC Canada

**Keywords:** Predictive markers, Depression, Quality control

## Abstract

Monitoring sleep and activity through wearable devices such as wrist-worn actigraphs has the potential for long-term measurement in the individual’s own environment. Long periods of data collection require a complex approach, including standardized pre-processing and data trimming, and robust algorithms to address non-wear and missing data. In this study, we used a data-driven approach to quality control, pre-processing and analysis of longitudinal actigraphy data collected over the course of 1 year in a sample of 95 participants. We implemented a data processing pipeline using open-source packages for longitudinal data thereby providing a framework for treating missing data patterns, non-wear scoring, sleep/wake scoring, and conducted a sensitivity analysis to demonstrate the impact of non-wear and missing data on the relationship between sleep variables and depressive symptoms. Compliance with actigraph wear decreased over time, with missing data proportion increasing from a mean of 4.8% in the first week to 23.6% at the end of the 12 months of data collection. Sensitivity analyses demonstrated the importance of defining a pre-processing threshold, as it substantially impacts the predictive value of variables on sleep-related outcomes. We developed a novel non-wear algorithm which outperformed several other algorithms and a capacitive wear sensor in quality control. These findings provide essential insight informing study design in digital health research.

## Introduction

Activity and sleep monitoring through ambulatory devices has become ubiquitous through use of commercial devices such as smartphones and smartwatches. Actigraphy, defined as activity and sleep monitoring through a research or medical-grade device worn on the body, has been in use for decades. It has been implemented in monitoring various populations, including individuals with sleep or biological rhythm disorders^1^, dementia^2^, and depression^3^, among others. Sleep and activity are the key monitoring targets for many disorders. For instance, sleep and activity are linked to quality of life^4^, mental, and physical health outcomes^5^. Activity and sleep disturbances have also been linked with increased risk of hypertension, diabetes mellitus, cardiovascular disease, coronary heart disease, obesity and mortality^6–8^.

Actigraphs are devices typically worn on the wrist, chest, or hip, which use motion sensing accelerometers to measure activity on one or three axes. One advantage of actigraphs is the potential for prolonged monitoring within the individual’s natural environment, which requires minimal effort on behalf of the device wearer and interaction with the device as compared to methods such as take-home questionnaires and ecological momentary assessment^1,9^. To date, the majority of studies have focused on periods of continuous data collection of 2 weeks or less^10^. Longer periods of data collection may be more informative, however, they have received less attention, likely because they require a more complex approach.

Detection of early signs of clinical changes is an important application of actigraphy. For instance, changes in sleep may be among the initial symptoms preceding the onset of a major depressive episode^11^. Actigraphy is therefore a promising tool to monitor early warning signs of depressive relapse. Actigraphy can be used to evaluate sleep parameters (e.g., total sleep time, sleep maintenance efficiency, wake after sleep onset), sleep timing (e.g., sleep onset time, time out of bed, mid sleep point), physical activity parameters (e.g., total activity counts, physical activity energy expenditure), circadian activity rhythms (e.g., cosinor analysis, which yields information about timing and intensity of activity), and other parameters. However, methods of actigraphy data collection and analysis, including collection parameters, devices used, data pre-processing, and variable extraction have not been standardized^1^.

Accurately and efficiently differentiating periods of wear from non-wear in actigraph data is a major challenge in actigraphy research. Ideally, participants should record off-wrist time in a dedicated log maintained throughout the duration of the study. However, this may be challenging in clinical populations, especially if participants suffer from difficulties with memory or attention, life stress, or other challenges that impair their ability to accurately record off-wrist time. As a consequence, automatic methods of detecting wear and non-wear periods have been developed. For instance, the ActiGraph GT9X Link is equipped with a capacitive sensor, which indicates whether the participant is wearing the device, based on the proximity of the device to skin, however, this wear sensor has technical issues, with non-wear being noted during times of apparent wear of the actigraph, as recorded by participants^12^. Consequently, the wear sensor substantially underestimates wear time compared to participant diaries, with a sensitivity of 93% but a specificity of 49%^13^. Additionally, there are several non-wear detection algorithms, though some of these were not developed to account for non-wear episodes during the night, or during sleep periods, and the majority of these algorithms were developed using data from actigraphs worn at the hip^14–16^. Importantly, the choice of pre-processing approaches, such as non-wear detection, sleep detection, and rules such as thresholds for what constitutes a valid number of days for actigraphy analysis can significantly impact outcomes in actigraphy studies^12,17^ Periods of non-wear may also be associated with outcomes of interest in mental health research, further supporting the importance of their accurate detection as part of studies of actigraphy in clinical populations.

The aim of this paper is to report on a standardized pipeline for quality control, pre-processing, and analysis of actigraphy data collected over an extended period of time, developed with the use of open-source packages.

## Methods

### Data collection

#### Study design

These actigraphy data were collected as part of the Wellness Monitoring for Major Depressive Disorder (Wellness Monitoring Study), a longitudinal observational study conducted by the Canadian Biomarker Integration Network in Depression (CAN-BIND), which aimed to identify predictive biomarkers of relapse of major depressive disorder (MDD) (ClinicalTrials.gov Identifier: NC02934334). The Wellness Monitoring Study used ambulatory monitoring to establish which variables can act as “warning signals” prior to a relapse of MDD. Several symptom domains were evaluated, including mood and anxiety symptoms, sleep, activity, biological rhythms, anhedonia, pain, quality of life, treatment compliance-related variables, speech characteristics and voice characteristics. The domains were assessed through different methods, including self-report questionnaires, clinician-rated assessments, audio recording of voice, and objective monitoring of activity, sleep and biological rhythms with actigraphy.

Participants were enrolled into the study if they had a diagnosis of MDD, responded to treatment for their most recent major depressive episode, and had a current MADRS score < 14 at baseline and screening visits, resulting in a total of 101 participants who completed a baseline visit. Following written informed consent, participants received a study-specific smartphone (LogPad®, ERT, Clario [formerly, PHT]) and wrist-worn actigraph, which were used for the duration of the study. Further information about the study sample is provided in the Supplementary Materials, including supplementary Figure 1 which describes participants in the Wellness Monitoring study.

Participants completed a screening visit, a baseline visit within 2 weeks of screening, and a minimum one-year observational phase (early withdrawal allowed). Most participants completed screening and baseline visits on the same day. During the observational phase of the study, participants completed in-person assessments every 8 weeks in addition to continuous ambulatory monitoring. Participants enrolled on a rolling basis and had variable lengths of follow up periods with target durations of at least 1 year since last patient enrolled.

At baseline, and subsequent 8-weekly follow-up visits, participants were assessed through an on-site electronic data collection device (the SitePad®) which recorded measures of depressive symptom severity, healthcare service use, and symptom severity. Additionally, participants completed self-report questionnaires through the Brain-CODE REDCap interface and provided blood samples, as well as a series of weekly self-reports, and biweekly speech and voice characteristics through the LogPad® device. Further information about the study inclusion/exclusion criteria, treatment and relapse is provided in the supplementary material. All procedures contributing to this work comply with the ethical standards of the relevant national and institutional committees on human experimentation and with the Helsinki Declaration of 1975, as revised in 2008. Study procedures were approved by local research ethics boards and all participants provided informed consent before study entry.

#### Data acquisition: raw actigraphy data

The Actigraph GT9X-BT Link® (ActiGraph, Penascola, Florida, USA) device was used to collect sleep, activity and biological rhythms parameters through the observational phase of the study. Study coordinators uploaded the data to the CentrePoint Study Admin System (http://www.actigraphcorp.com/product-category/study-admin/) and monitored adherence during in-person visits. CentrePoint is a cloud-based technology platform developed by Actigraph, which preserved data integrity, as well as network security, availability, and standards compliance. The GT9X Link contains a capacitive touch wear sensor^18^.

**Figure 1:**
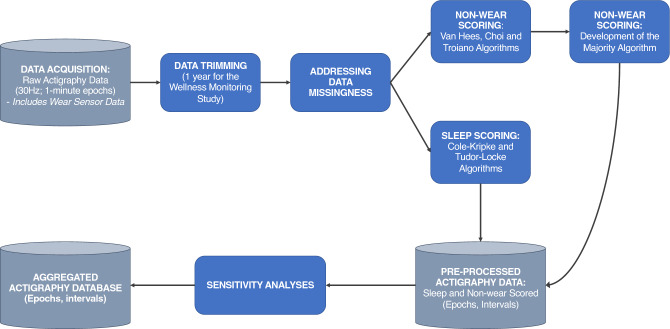
Pre-processing Pipeline Summary. Raw actigraphy data were acquired at 30 Hz and pre-processed to minute epochs, where activity data, a timestamp and corresponding wear sensor data were extracted. These data were trimmed, and data missingness patterns were addressed. Non-wear scoring (using the van Hees, Choi and Troiano algorithms) and sleep–wake scoring (using the Cole-Kripke and Tudor-Locke algorithms) were completed. A novel non-wear scoring method was developed, combining data from the van Hees, Choi and Troiano algorithms, with the wear sensor data (the Majority algorithm). Next, sleep and non-wear data were combined at the interval and epoch levels. A sensitivity analysis was performed to assess optimal threshold for overlap of sleep with non-wear intervals, yielding a final aggregated actigraphy database at the epoch and interval levels.

Participants were instructed to wear the GT9X Link® device 24 h per day for the entire duration of the study, and received a charging dock and USB cable to charge the device from home. Data were collected at 30 Hz on the non-dominant wrist. At each in-person visit, data were extracted to the CentrePoint system by study coordinators. Data from the CentrePoint system were transferred to OBI’s Brain-CODE platform at the completion of the study. Data were first extracted as raw .gt3x files, at intervals corresponding to occasions on which data were uploaded. Data were additionally aggregated into minute-by-minute epochs, as one .csv file for each participant, and were initially sleep scored using the Cole-Kripke algorithm^19^ (Fig. 1: Raw Actigraphy Data).

Raw actigraphy data provided information about the direction and orientation of the actigraph, while count data only provided information about the amount of movement. Count data aggregated by epoch are traditionally used as the basis of calculating sleep^[20]^and energy expenditure parameters^20^, as well as non-wear, while more recent actigraphy processing methods use raw data^16,21^.

### Data processing and analysis

#### Summary

Figure 1 shows a summary of the automated data pre-processing pipeline, as executed in R Statistical Software (v 4.0). As part of this pre-processing pipeline, we assessed data missingness and scored sleep and wake for minute-by-minute epochs using the Cole-Kripke^19^ and Tudor-Locke^22^ algorithms. Next, we tested the accuracy of four methods of non-wear detection: (1) the built-in wear sensor available in this actigraph model; scored the minute-by-minute epoch data using the (2) Choi^14^ and (3) Troiano^15^ algorithms; and (4) used the raw 30 Hz actigraphy data for scoring using the van Hees algorithm^23^. From these four methods, we created a new non-wear scoring algorithm (the majority algorithm), and conducted visual quality control of this majority algorithm (See *“Non-wear detection”* section below). Next, we combined the sleep intervals with non-wear intervals, and conducted sensitivity analyses to assess the influence of valid day selection and percentage of overlap between non-wear and sleep on the relationship between sleep variables and the main outcome measure of this study – the Montgomery-Åsberg Depression Rating Scale (MADRS)^24^, which was collected at each in-person visit.

#### Data trimming

An important step in data pre-processing is to trim the data including only data that will be used for analysis. For instance, in case of withdrawal from the study, participants may have worn the actigraph (or the actigraph may have collected data) until it is returned to the lab, at a later date than the official withdrawal date from the study. Additionally, researchers may only be interested in analyzing a specific portion of the collected data, in which case data trimming is also necessary. In the Wellness Monitoring Study, data were trimmed to 1 year of collection, and data that extended following the participant’s enrollment or collected due to configuration error prior to enrollment in the study were trimmed based on study enrolment dates. Duplicate rows were removed (Fig. 1: Data Trimming).

It is important to ensure that data for all dates were accounted for, including periods of missing data, if such paradata were to be recorded or reported. Paradata refers to administrative data that were obtained during the process of collection, management and treatment of actigraphy data^25^. If a participant was asked to wear multiple actigraph devices throughout the duration of the study, the periods of overlap must be correctly accounted for, and the correct data interval should be used. We maintained accurate paradata of the rows that were removed, and the number of missing minutes per day, per participant, which will be stored and made available with the pre-processed data.

#### Sleep scoring

Minute-by-minute epoch data were scored for sleep and wake using the Cole-Kripke and Tudor-Locke algorithms deployed in the *actigraph.sleepr* package (https://github.com/dipetkov/actigraph.sleepr), which is an open-source implementation of the ActiLife software’s sleep and non-wear detection algorithms (Fig. 1: Sleep/Wake Scoring: Cole-Kripke and Tudor Locke Algorithms). From this analysis, epoch-based scoring of minute epochs and sleep intervals were obtained. Sleep intervals were characterized by the following variables: sleep maintenance efficiency (SE, %), sleep duration (mins), activity counts, non-zero epochs, total sleep time (TST, mins), wake after sleep onset (WASO, mins), number of awakenings, movement index, fragmentation index, sleep fragmentation index, sleep onset time (HH:MM:SS), time out of bed (HH:MM:SS), number of one minute sleep intervals, mean mid sleep time ([time out of bed – sleep onset time]/2), average awakening (mins). Fragmentation index is calculated as a percentage of sleep periods that last 1 min compared to number of periods of sleep during the sleep period. Movement index consists of the percentage of epochs during the sleep period where y-axis counts were larger than zero. Sleep fragmentation index is the sum of the movement index and fragmentation index^26^.

#### Non-wear scoring

In the Wellness Monitoring Study, we used the wear sensor embedded in the Actigraph GT9X Link, in addition to the Troiano, Choi and van Hees algorithms to detect non-wear. The Troiano and Choi algorithms were chosen due to their wide use, ease of implementation, and availability through the ActiLife software. The van Hees algorithm was chosen due to its superior performance in Syed and colleagues’ study^27^, and ease of implementation. The Troiano and Choi algorithms use epoch-aggregated count data^14,15^. The Troiano algorithm defines non-wear intervals as 60 or more consecutive minute epochs with no activity, allowing for 1 or 2 min of counts of 0 to 100^15^. Since this algorithm is prone to classifying sedentary activity as non-wear time, Choi and colleagues proposed a modified algorithm where non-wear was classified as intervals of at least 90 min with consecutive minute epochs of no activity. Intervals of 1 or 2 min with non-zero counts would not change this classification, if there was no activity 30 min before or after that interval^14^. Newer approaches such as the van Hees algorithm use raw data^16^. Van Hees’ algorithm is based on raw data, where a period is deemed to be non-wear when the standard deviation of movement is lower than 3.0mG (1mG = 0.00981 m/s^2^) or the value range is lower than 50 mg for at least 2 of 3 axes for a given 30-min period^16,23^. These approaches are useful to detect longer periods of non-wear, however, shorter periods of non-wear (e.g., taking the actigraph off for showers), will not be detected.

The capacitive sensor on the Actigraph GT9X Link provided epoch-aggregated non-wear detection at the minute level. The capacitive sensor consists of a metallic plate. Based on the concept of capacitive coupling, the sensor charges more quickly when it is in closer proximity to our bodies. The sensor therefore measures the amount of time that the capacitor uses to charge, and therefore allows estimation of non-wear^28^. Troiano^15^ and Choi^14^ algorithms were used to score the activity (motion) data from csv files containing minute-by-minute data using the *actigraph.sleepr* package (Fig. 1: Non-wear Scoring: Choi and Troiano Algorithms). Additionally, non-wear scoring was performed on the raw data gt3x files using the van Hees algorithm through the *GGIR* package^23^. While using this package, we specified a 5 s window for calculating acceleration and angle, 900 s for the epoch length to calculate non-wear and signal clipping, and 3600 s for the window of wear detection (Fig. 1: Non-wear Scoring: Van Hees Algorithm). Agreement between algorithms during each epoch was evaluated through minute-by-minute overlap of non-wear detected by the different algorithms and the wear sensor. Additional information about data processing is provided in the Supplement.

##### Development of a novel non-wear algorithm: the majority algorithm

A novel non-wear algorithm, the majority algorithm, was developed by calculating the percentage of overlap between the wear sensor, Troiano, Choi and Van Hees algorithms in each minute epoch (Fig. 1: Non-wear Scoring: Development of the Majority Algorithm). If 3 or 4 of the 4 methods of detection indicated that a minute epoch should be classified as non-wear, this minute epoch was classified as non-wear. As the Choi algorithm is an updated version of the Troiano algorithm, we compared the performance of a 4-method version of the majority algorithm (which combined the wear sensor, Troiano, Choi and van Hees algorithms) to a 3-method version of the majority algorithm (which only used the wear sensor, Choi and van Hees algorithms). For the 3-method version, if 2 or 3 of the 3 methods of detection indicated that a minute epoch should be classified as non-wear, this minute epoch was classified as non-wear. To validate the use of this algorithm, we performed visual quality control to evaluate performance of the majority algorithm in a subset of participants. We selected a majority of these participants based on their relapse status, as this was the major outcome in the Wellness Monitoring Study (see Supplementary Material). Each participant file was reviewed day-by-day, where false non-wear detection was identified by one or two trained independent scorers (see Supplementary Material for further details). Accuracy, positive predictive value, sensitivity and specificity statistics were calculated for epoch-level data for each of the 5 algorithms (Choi, Troiano, van Hees, majority (4), and majority (3)) and the wear sensor, as compared to visual quality control at the day level. As 6 of the participant data files were scored by 2 scorers, we averaged the results of the accuracy, positive predictive value, sensitivity and specificity statistics for these participants for the outputs of the algorithms compared to visual quality control. To test the difference in performance of the algorithms, we fitted mixed linear models, with day-level performance statistics as dependent variables and algorithm*day as the independent variables using the *lme4* package. We compared the performance of the different algorithms using estimated marginal means of the models, with a Tukey correction for multiple comparisons using the *emmeans* package. Inter-rater reliability (Cohen’s kappa) was calculated.

#### Addressing data missingness

Some analytic procedures require complete data. Data missingness can be classified as missing completely at random (MCAR), meaning that missing data are missing independently of observed or missing data. This type of missingness does not cause bias, despite increasing standard error. Missing at random (MAR) data occur when the mechanism of missingness is a partial result of the observed data, and if the mechanism of the missing data is a result of the missing data, this indicates the data are not missing at random (NMAR)^29^.

It is plausible that participants’ non-wear may correspond with periods of relapse of depression, which is the key outcome measured in the Wellness Monitoring Study, indicating that these data are likely not MAR or MCAR. Additionally, summary statistics regarding non-wear can be used in modeling outcomes during the analysis stage. Therefore, we intend to use missing data as part of our modelling approach, where variables describing non-wear and missingness will be included in predictive models for mental health outcomes.

At the epoch level, we used the average day imputation method, where missing data are imputed by an average of the values collected during the same time period that has missing data (for instance, if data are missing from 7:00 to 7:15, this algorithm will create an average for that missing interval based on the data that were collected)^30^. To perform this average day imputation, we used a window of 7 days (i.e., 3 days prior to and 3 days following the day with missing data). We did not impute full days of data – only days with partial missing data were imputed. In this study, data could have been missing as a result of non-wear (based on the majority (3) algorithm) or as a result of data not being collected for the period (Fig. 1: Addressing Data Missingness).

Spearman correlations were applied to assess the relationship between depressive symptoms according to the MADRS and data missingness or non-wear patterns. As the data for sleep and depressive symptoms were assessed at different frequencies, we aggregated these data by creating an average of each sleep variable.

#### Sensitivity analyses

Many studies in actigraphy literature use filtering approaches, where days are only considered valid if the actigraph is worn over a certain number of hours for each day^31^. This threshold has not been standardized, though the most commonly used threshold is 10 h or more of available data in a day^31^, for the day to be considered valid. A sensitivity analysis was conducted to test influence of non-wear on the relationship between sleep and MADRS scores, the main symptom outcome measure in this study. This sensitivity analysis consisted of two components: (1) number of valid hours of data per day for the day to be considered valid and (2) overlap of the sleep interval with non-wear, and how these components influenced the relationship between sleep variables and depressive symptoms (Fig. 1: Sensitivity Analyses).

First, this sensitivity analysis used hourly thresholds starting from > 6 to 24 valid hours per day of analysis for the relevant sleep interval to be included in the analysis, as well as all collected data. The second component of the analysis selected several thresholds for excluding intervals of sleep based on overlap with non-wear. Overlap of sleep with non-wear intervals was calculated for each sleep interval, first by generating the number of non-wear minutes in each sleep interval, and subsequently calculating percentage of non-wear minutes per duration of the sleep interval. Thresholds were tested in 10% intervals, ranging from < 10% overlap to up to 100% overlap. Sleep intervals exceeding a given threshold (e.g. > 80% overlap) were excluded from analysis for each iteration of this analysis. Since MADRS scores were obtained every 8 weeks for the duration of the study, and at each relapse verification visit, we averaged sleep values across each 8-week epoch. For each combination of thresholds, we conducted mixed linear modeling with the following variables, following standardization, as fixed-effects variables used to model of MADRS score: sleep variables (SE, duration, activity counts, non-zero epochs, TST, number of awakenings, movement index, fragmentation index, sleep onset time, out of bed time, number of one minute sleep intervals, average awakenings), time since study enrolment and number of missing or non-wear minutes, and participant ID as a random intercept . We evaluated 190 combinations of overlap threshold and valid day selection, and chose the threshold combination with the lowest marginal R^2^ ^32^.

### Statistical software

All analyses were implemented in R statistical software (v. 4.0).

## Results

### Collected and missing data

Summary statistics outlining collected data and missingness in the Wellness Monitoring Study are outlined in Table 1, describing missingness due to a lack of data collection at the minute epoch level. Overall, participants were observed for a total of 31,175 days, amounting to 44,891,400 rows (minute epochs) of data. Overall, 36,600,320 rows of data were collected (25,416.89 days), with 18.47% or 8,291,080 rows of data missing across the period of data collection (5,757.69 days). If aggregated at the participant level, each participant had between 0.11 to 100% of data missing. A total of 95 participants had available actigraphy data, and completed 8 weeks of data collection. By 26 weeks of data collection, 84 participants (88.4%) continued data collection, and 73 participants (76.8%) remained by the 52^nd^ week of data collection.

**Table 1:** Data missingness in the wellness monitoring study.

	Total	Mean ± SD per participant	Range per participant		
N days of data collected	31,175	328.16 ± 84.51	57–366		
N rows of data collected	44,891,400	472,541.05 ± 121,704.76	82,080–527,100		

	Total	Mean ± SD per participant	Mean ± SD per participant per day	Range per participant	Range per participant per day
N missing rows of data	8,291,080	87,274.53 ± 107,447.15	287.15 ± 349.02	581–502,354	1.59 ± 1440

	% of total	% ± SD per participant	Range per participant		
% missing rows of data	18.47%	19.94 ± 24.24	0.11–100%		

### Non-wear detection

#### Summary of non-wear according to different algorithms

**Figure 2:**
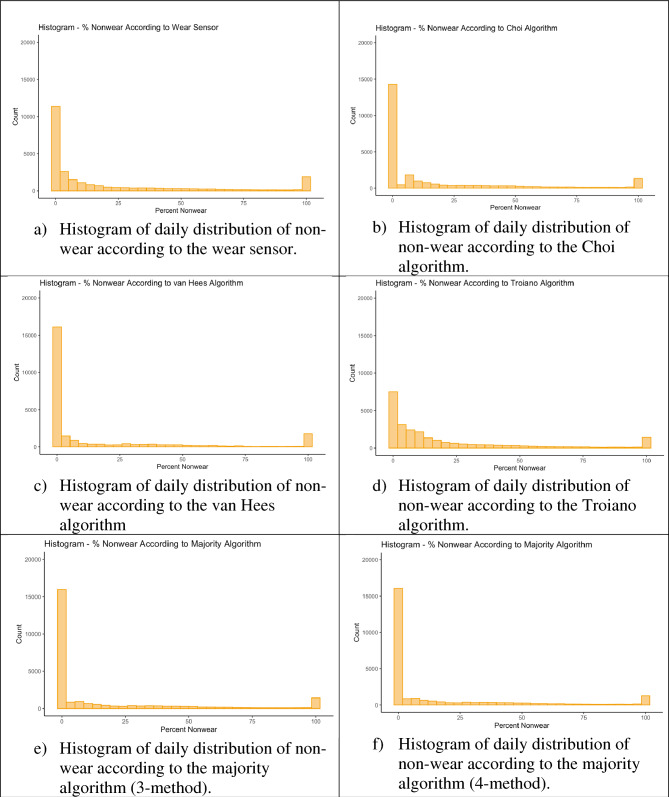
Distribution of non-wear per day according to different methods of non-wear detection.

Table 2 displays non-wear statistics obtained from the non-wear detection methods throughout the study. At the day level, according to the 3 non-wear algorithms, there was a mean of 12.55 to 16.74% of data missing overall throughout the study, whereas the wear sensor detected 16.29% of non-wear throughout the study. At the participant level, where mean statistics were aggregated per participant, each participant had 12.43 to 16.62% of non-wear. Figure 2 shows the distribution of non-wear per day as detected by the different methods.

**Table 2:** Summary of non-wear in wellness study according to different methods of detection.

	Median	Mean ± SD	Range	Median Excluding Missing Data)	Mean/SD (Excluding Missing Data)	Range (Excluding Missing Data)
Participant-level statistics (Aggregated per Participant) (n = 95 participants)						
% Non-wear: GT9X-Link Wear Sensor	13.04	16.32 ± 11.40	0–55.44	17.90	22.39 ± 16.16	3.83–86.19
% Non-wear: Choi	9.66	13.78 ± 12.71	0–56.60	12.78	20.28 ± 19.21	0.94–85.54
% Non-wear: Troiano	12.34	16.62 ± 12.79	0–58.64	18.63	23.67 ± 18.94	1.58–86.39
% Non-wear: van Hees	7.11	12.43 ± 12.62	0–55.35	9.04	18.56 ± 19.29	0.52–85.53
% Non-wear: Majority algorithm	7.79	12.50 ± 12.46	0–55.75	9.71	18.63 ± 19.06	0.80–85.66
% Non-wear: Majority algorithm (3)	8.41	12.73 ± 12.54	0 – 55.97	9.82	18.95 ± 19.20	0.80–85.74
Day-level statistics (Aggregated Across Entire Study) (n = 31,175)						
% Non-wear: : GT9X-Link Wear Sensor	0.90	16.29 ± 28.85	0–100	3.19	20.08 ± 31.00	0–100
% Non-wear: Choi	0.00	13.86 ± 26.65	0–100	0.00	17.03 ± 28.78	0–100
% Non-wear: Troiano	4.86	16.74 ± 26.65	0–100	8.13	20.53 ± 28.34	0–100
% Non-wear: van Hees	0.00	12.55 ± 26.98	0–100	0.00	15.42 ± 29.32	0–100
% Non-wear: Majority algorithm	0.00	12.58 ± 26.24	0–100	0.00	15.48 ± 28.47	0–100
% Non-wear: Majority algorithm (3)	0.00	12.82 ± 26.60	0–100	0.00	15.77 ± 28.85	0–100

#### Overlap of non-wear detection methods

Next, we assessed overlap of non-wear detected by the different algorithms and the wear sensor, finding a high proportion of overlap between all non-wear algorithms (91.55 ± 14.96%) at the day level across all participants. However, overlap with the wear sensor was lower, with a total of 79.32 ± 27.71% overlap of all methods of wear detection (Table 3). Additionally, this overlap of non-wear detection methods did not substantially change over time, as indicated by Figure S3c.

**Table 3:** Mean and standard deviation daily percent overlap of non-wear in wellness study according to different methods of detection – (Day level) (n = 31,175).

Missing	5,536			
% Agreement Between All Methods of Detection (excluding Troiano)	82.35 ± 28.19			
% Agreement Between All Methods of Detection (Including Troiano)	79.32 ± 27.71			
% Agreement Between All Algorithms (Troiano, Choi, van Hees)	91.55 ± 14.96			

	Wear sensor	Choi algorithm	Troiano algorithm	Van Hees algorithm
Wear Sensor	1			
Choi Algorithm	85.25 ± 25.61	1		
Troiano Algorithm	82.94 ± 24.90	95.82 ± 5.67	1	
Van Hees Algorithm	83.96 ± 27.80	95.12 ± 14.30	92.10 ± 14.68	1

Median daily percent overlap of non-wear in wellness study according to different methods of detection				
% Agreement Between All Methods of Detection (excluding Troiano)	94.72			
% Agreement Between All Methods of Detection (Including Troiano)	90.42			
% Agreement Between All Algorithms (Troiano, Choi, van Hees)	95.42			

	Wear sensor	Choi algorithm	Troiano algorithm	Van Hees algorithm
Wear Sensor	1			
Choi Algorithm	96.88	1		
Troiano Algorithm	93.04	98.61	1	
Van Hees Algorithm	96.53	100	95.63	1

#### Development of a novel non-wear algorithm: the majority algorithm

Table 4 shows performance of the 3-method and 4-method non-wear majority algorithms compared to the other methods of non-wear detection.

Detailed visual quality control was conducted to test the performance of the 4-method majority algorithm on data from 19 participants (20% of the total sample), for a total of 4,600 days, or 6,624,026 rows. Figure S2a shows an example of the visualization used to conduct quality control for the majority algorithm. Most selected participants (n = 15) were chosen from based on their status as relapsers at some point during the study, and additional participants were selected from the non-relapser group to strengthen the validity of this evaluation (n = 4). Inter-rater reliability measured through Cohen’s kappa was κ = 0.94, indicating near perfect inter-rater reliability^33^, calculated from 1,991 days of data obtained from 6 participants assessed by 2 raters.

**Table 4:** Performance of non-wear detection methods in visual quality control.

	Wear Sensor Mean (SD)	Choi Algorithm Mean (SD)	Troiano Algorithm Mean (SD)	Van Hees Algorithm Mean (SD)	4-method Majority Algorithm: Wear Sensor, Choi, Troiano, Van Hees Mean (SD)	3-method Majority Algorithm: Wear Sensor, Choi, van Hees Mean (SD)
Total Rows	6,702,388	6,702,388	6,702,388	6,624,026	6,624,026	6,624,026
Total Days	4,654.44	4,654.44	4,654.44	4,600.02	4,600.02	4,600.02
Missing	1,823,252	1,823,252	1,823,252	1,901,614	1,901,614	1,901,614
Accuracy	0.8839 (0.2722)	0.9816 (0.0564)	0.9609 (0.0683)	0.9866 (0.0474)	0.9884 (0.0526)	0.9887 (0.0517)
Positive Predictive Value	0.6197 (0.4703)	0.9101 (0.2762)	0.6723 (0.4528)	0.9515 (0.2063)	0.9665 (0.1711)	0.9641 (0.1767)
Sensitivity	0.9444 (0.2225)	0.9617 (0.1707)	0.9823 (0.1067)	0.9289 (0.2013)	0.9608 (0.1609)	0.9592 (0.1620)
Specificity	0.9154 (0.2326)	0.9885 (0.0491)	0.9632 (0.0717)	0.9967 (0.0370)	0.9982 (0.0125)	0.9972 (0.0280)

Mean and SD of the algorithm performance statistics were calculated at the day level.

SD = standard deviation.

A visualization of the comparative performance of the wear sensor, Choi, Troiano, van Hees and majority (3- and 4- method) algorithms can be found in Supplementary Figure S2b, and results from models comparing algorithm performance statistics can be found in Supplementary Tables S1 and S2. Between the wear sensor, Choi algorithm, Troiano algorithm, van Hees algorithm and majority algorithms (3-method and 4-method versions), the majority algorithms had the best overall performance. The majority algorithms had significantly better accuracy than the wear sensor, Choi and Troiano algorithms (3-method: 0.9887; 4-method: 0.9884; wear sensor: 0.8839; Choi algorithm: 0.9816; Troiano algorithm: 0.9609). The van Hees (0.9866) and majority algorithms had similar accuracy, though the van Hees algorithm’s accuracy did not significantly differ from the Choi algorithm. The majority and van Hees algorithms had performed significantly better than all other methods in specificity (4-method: 0.9982; 3-method:0.9972; van Hees algorithm: 0.9967; Choi algorithm: 0.9885; Troiano algorithm: 0.9632; wear sensor: 0.9154) and PPV (4-method: 0.9665; 3-method: 0.9641; van Hees algorithm: 0.9515; Choi algorithm: 0.9101; Troiano algorithm: 0.6723; wear sensor: 0.6197). Finally, the Troiano algorithm significantly outperformed all other algorithms in terms of sensitivity, followed by the Choi and majority algorithms (Troiano algorithm: 0.9823; Choi algorithm: 0.9617; 4-method: 0.9608; 3-method: 0.9592; wear sensor: 0.9444; van Hees algorithm: 0.9289). The wear sensor had the poorest performance in non-wear detection. Notably, these statistics only capture visually noted intervals of non-wear, which were typically over the length of an hour. Since the 3- and 4-method majority algorithms had comparable performance, which exceeded the single algorithms in accuracy, we used the 3-method majority algorithm in the remainder of our analyses.

**Figure 3:**
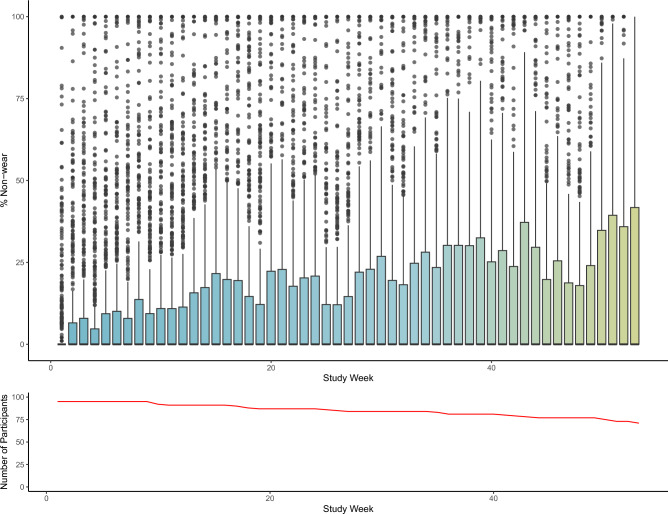
Percentage of non-wear detected using majority algorithm across study duration.

In line with previous investigations^34^, non-wear increased with time since baseline, and variability in non-wear increased with time since baseline, as data from fewer participants were available (see Fig. 3, mean of 4.8% in the first week to 23.6% at the end of 12 months of data collection).

### Managing data missingness

#### Addressing data missingness in the Wellness Monitoring Study

When we combined non-wear scoring with sleep interval scoring, there were, expectedly, periods of overlap between these intervals. We used Spearman correlations to see whether there was a relationship between the main clinical outcome (depressive symptoms according to the MADRS) and data missingness or non-wear patterns. Depressive symptoms according to the MADRS did not correlate with data missingness (rho = − 0.04), nor with non-wear patterns according to any of the methods of non-wear detection (rho = − 0.03 to 0.02) (Figure S3).

#### Sensitivity analyses

Next, we conducted a sensitivity analysis of the influence of the overlap of sleep intervals with non-wear intervals, and influence of valid day criteria. We tested 200 thresholds for excluding sleep intervals which overlapped with non-wear, and their combination with thresholds of number of hours of data per day for the day to be considered valid, and assessed whether these thresholds impacted the relationship of individual sleep metrics with depressive symptoms. Overlap thresholds were tested in 10% increments, ranging between < 10% overlap and 100% overlap. Valid day thresholds were tested in hourly increments ranging from all collected data, > 6 valid hours to 24 valid hours. See Supplementary Figure S5 for an illustration of this thresholding approach (Table 5).

Altogether, there were 30,093 sleep intervals available for evaluation, and a maximum of 12,438 sleep intervals were excluded through the non-wear percentage threshold approach. There were 515 instances of MADRS observations across the study for 94 participants with valid actigraphy data. The threshold combination of > 20 valid hours and up to 30% overlap between sleep and non-wear intervals, was chosen based on the highest marginal R^2^ value for mixed linear models (See Table 5). This yielded 22,853 total sleep intervals.

**Table 5:**
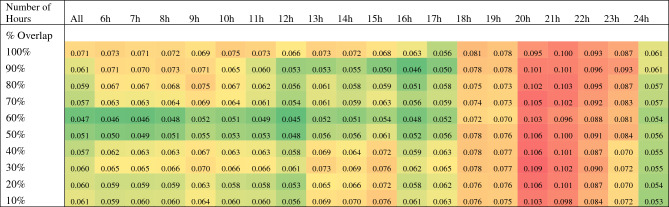
Sensitivity analysis results: combinations of non-wear thresholds based on 24-h non-wear and % overlap between sleep intervals with non-wear.

Marginal R^2^ Values Modeling Montgomery-Åsberg Depression Rating Scale Scores as a Function of Sleep Variables in Mixed Linear Models.

## Discussion

In this study, we present a data-driven pre-processing pipeline for a long-term actigraphy study using the example of the Wellness Monitoring Study which lasted over the course of 12 months of continuous data collection. This study provides a guideline for future digital health research using large, longitudinal actigraphy datasets. Importantly, a novel algorithm for non-wear detection, the *majority algorithm* was developed, which involved an extensive visual quality control procedure. The *majority algorithm* significantly outperformed the use of single common non-wear detection methods in terms of accuracy, specificity and positive predictive value, including the GT9X Link wear sensor, the Choi, and Troiano algorithms, and outperformed the van Hees algorithm in sensitivity. A key advantage of the majority non-wear algorithm is that it is relatively easy to implement and will be useful for other models of ActiGraph devices, which also use the capacitive sensor for detecting skin conductance and non-wear. Moreover, this algorithm was developed using open-source packages that are widely available to the public. We found that the wear sensor had the worst performance compared to the algorithms that were calculated, though it was likely able to detect short periods of non-wear that the visual quality control procedure was likely unable to detect, as the visual quality control procedure was not able to verify short non-wear periods. Additionally, the non-wear algorithms were only able to capture intervals of non-wear that were typically over the length of an hour. Our findings of inconsistency in wear sensor performance are similar to both Pulakka and colleagues’ and Arguello and colleagues’, who also witnessed off-wrist time shown by the wear sensor during apparent wear time, and poor sensitivity of the wear sensor^12,13^.

As expected, compliance with actigraph wear decreased progressively over the course of the year, from a mean of 4.8% at the beginning of the study, to a mean of 23.6% by the end of the year-long study. To date, the majority of studies using actigraphy have used significantly shorter periods of data collection^10^, with some studies reporting wear compliance through periods of 16 weeks to 1 year^34–36^. In a 16-week longitudinal actigraphy study, Thurman and colleagues found 95.1% compliance with actigraphy measurements, with no changes over time^34^. In contrast, in a 6-month longitudinal study of pain in patients with sickle cell disease, of the possible 6 months of data collection, participants completed a median of only 85 days of actigraphy data, with a range of 7 to 179 days of data collected, as a result of compliance and technical issues^35^. A feasibility actigraphy study of 8 participants followed for a total of 150 weeks with the aim of predicting relapses in bipolar disorder had a total of 30% of data missing^36^. This suggests that there is a range of compliance in studies with actigraphy devices, where longer study duration is associated with lower compliance. We interpret the approximately 70% completeness in actigraphy data obtained in 95 participants with major depression over a 12-month study as positive.

An important strength of our methods study is the amount of data available to us through the longitudinal, naturalistic design of the Wellness Monitoring Study. This unique, longitudinal dataset showed that non-wear increases over the course of a year, though a substantial proportion (n = 59) of participants continued to wear their actigraph until the end of the year mark. Moreover, we were able to address challenges of data pre-processing consistency, by providing a pre-processing pipeline for data extraction, trimming, sleep and non-wear scoring, combining sleep and non-wear intervals, and non-wear threshold selection. In a real-world application, where actigraphs are used to detect, for instance, early signs of relapse, or subtle changes in physical activity, longitudinal data spanning a substantial period of participants’ lives may be used as an early signal.

We found that a threshold of 20 or more valid hours per day combined with 30% or less overlap of sleep intervals with non-wear yielded the best performance of sleep variables as an explanatory variable for depressive symptoms. The findings of our sensitivity analyses support the importance of selecting an appropriate valid day and/or percentage overlap of sleep interval with non-wear criteria in order to obtain stable estimates of the influence of sleep variables on depressive symptoms. This finding is in line with previous studies^12,17^, which indicated that pre-processing choices, such as selecting valid day filtering rules impact the influence of physical activity on outcomes. We suggest that future studies control for non-wear based on similar considerations, accounting for the influence of these non-wear thresholds on outcomes.

### Limitations

One limitation of this study is the lack of ability of the ActiGraph GT9X Link to adequately detect sleep onset latency without use of a sleep diary. This type of actigraph provides an output of “0” for each of the instances of this value if a sleep diary is not used. This likely means that our estimates of sleep maintenance efficiency were possibly overestimated. Notably, the participants in our study were diagnosed with MDD, and may not reflect the patterns of activity in the general population, and may have a different propensity to remove the actigraph (for instance, during relapse) compared to the general population.

The *majority algorithm* should be further validated in an independent dataset which is able to provide the actual accurate periods of non-wear, as opposed to visual quality control through a sleep diary or some other measure. Having a sleep diary would allow us to verify the periods of sleep accurately as well, however, in a dataset of this size, with over 31,000 days of data collected, comparing actigraphy data with data from several thousand of sleep diaries would be a significant challenge.

### Future directions

Recently, Syed and colleagues trained a deep convolutional neural network algorithm to detect non-wear from raw data by attempting to identify the instance of the hip-worn actigraph being removed and replaced, providing a more precise non-wear algorithm, which performed with high positive predictive value, sensitivity and F1 scores (all above 0.99). One drawback to this algorithm is the need to resample to a frequency of 100 Hz, indicating that data points that do not exist must be interpolated and the effects of resampling on the integrity of the data have not been explored^37^. Additionally, future studies should investigate the influence of actigraph non-wear time with clinical characteristics of MDD, including relapse, mood symptom worsening, behavioural inhibition, and psychosocial functioning.

## Conclusions

This study provides a standardized pre-processing pipeline for a longitudinal actigraphy study, in which data were collected continuously in 95 participants for one year. A novel non-wear algorithm was proposed which outperformed several single algorithms and a capacitive wear sensor in an intensive quality control procedure. Compliance with actigraph wear decreased over time, and sensitivity analyses demonstrated the importance of selecting pre-processing thresholds, as they substantially impacted the predictive value of variables on our main clinical outcome.

### Supplementary Information

Supplementary Information.

## Data Availability

CAN-BIND and the CAN-BIND Wellness Monitoring study are open science. Data will be released through Ontario Brain Institute’s Brain—CODE platform, which provides the ability to capture and manage data, and enables researchers to share their data, maximizing data discovery (https://www.braincode.ca).
