# Supplementary material for: A standardized workflow for long-term longitudinal actigraphy data processing using one year of continuous actigraphy from the CAN-BIND Wellness Monitoring Study
Source: Sci Rep. 2023 Sep 15;13:15300. doi: 10.1038/s41598-023-42138-6 (PMC10504311; doi:10.1038/s41598-023-42138-6)
Supplement: Supplementary file 1 — Supplementary Information. [file 41598_2023_42138_MOESM1_ESM.docx]

## Supplementary Material - Methods

Inclusion criteria for participants were 1) being outpatients in the age range of 18 to 65; 2) meeting criteria for a major depressive episode, with past or recurrent MDD, as determined through the Mini International Neuropsychiatric Interview (MINI); 3) response to treatment (monotherapy or combined therapy) for MDD during the current or most recent major depressive episode; 4) having a total score of 14 or less on the Montgomery-Åsberg Depression Rating Scale (MADRS) at baseline and screening visits; 5) willingness and ability to complete self-reported assessments on a specialized smartphone used for the study (LogPad®), which required fluency in English; 6) willingness to wear an actigraph device throughout the duration of the study.

Exclusion criteria were as follows: 1) a primary Axis I diagnosis other than MDD; 2) lifetime diagnosis of either bipolar I or II disorder, MDD with psychotic features, schizophrenia, or schizoaffective disorder; 3) a significant Axis II diagnosis, such as borderline or antisocial personality disorder; 4) high risk of suicide, as determined through clinical judgment; 5) 6-month history of moderate or severe substance or alcohol use prior to the screening visit; 6) presence of significant neurological disorder, head trauma, or unstable medical conditions.

Participants continued their treatment regimen upon entry in the study as prescribed by the study psychiatrist throughout the study. Treatment adjustments were permitted as clinically indicated, and were documented through a case report form.

At the discretion of the study psychiatrist, medications for stable medical conditions, as well as vitamins, supplements, family planning methods, and over-the-counter medication for pain were permitted. These were disclosed by the patient and recorded in a note to file for the purpose of data analysis.

Criteria for relapse included participants’ having a MADRS score of >= 22 at one of the study visits, and confirmed during a relapse verification visit; antidepressant dose increase/addition; elevated QIDS scores; and judgment of clinicians who were blinded to the identity of the participants.

***Actigraphy***

Sleep onset latency was calculated by the algorithm, though it appeared as a value of 0 for the entire dataset. Therefore, this variable could not be used as part of the analyses. The data collected from the Actigraph GT9X did not permit the calculation of sleep onset latency due to that the Tudor Locke algorithm for estimating the start and finish of a sleep interval requires 5 consecutive epochs to be indicated as sleep, in order to mark an interval as a sleep interval. This is described by ActiGraph (https://actigraphcorp.my.site.com/support/s/article/What-does-the-Detect-Sleep-Periods-button-do-and-how-does-it-work).

In order to combine non-wear and sleep data from the different algorithms, we extracted the non-wear scores from the output files produced by the *GGIR* package, and mapped them to the epoch-aggregated minute-by-minute files. For each algorithm (i.e., Choi, Troiano and van Hees) we obtained a file which summarized the non-wear intervals and scoring mapped to the minute-by-minute epoch-aggregated files.

**Development of a novel non-wear algorithm**

We created a visualization of the data collected each day for these participants, where we included the following, based on 1-minute epochs: 1) vector magnitude (i.e., raw acceleration data), 2) sleep scoring (using the Cole-Kripke algorithm), 3) sleep interval (using the Cole-Kripke and Tudor-Locke algorithms), 4) wear according to the wear sensor, 5) wear according to the Choi algorithm; 6) wear according to the Troiano algorithm; 7) wear according to the van Hees algorithm; 8) wear according to the majority algorithm 9) a summary row indicating percentage of non-wear, as indicated by these 4 methods of detection.

**Visual Quality Control**

The goal of visual quality control was for scorers to provide a file which indicated intervals of true non-wear for each participant. Scorers used the visualization described above and visualized in Supplementary Figure S2a as a primary source of information, and used the non-wear interval file and raw epoch-based file for reference in order to indicate where the true non-wear intervals occurred. Independent scorers were trained to distinguish intervals where the combination of activity, sleep-wake scoring, and non-wear scoring methods indicated non-wear. Each participant’s files were reviewed day-by-day and true non-wear intervals were generated through this process.

Independent scorers (authors AS, SH, CM, PKL, ARD, AM, KH) were trained during an initial videoconferencing session by an actigraphy expert (AS). This session was recorded and used for reference to the scorers. During this training session, one of the participants’ records was reviewed, including 1) the data visualization described above, 2) a csv file containing the time intervals for non-wear as assessed through the majority (4) algorithm, where a start time and end time was provided for each interval. Finally, 3) a csv file containing the raw actigraphy data aggregated into 60 second epochs was provided to scorers, where the following information was available for each minute of data collection for each participant: timestamp; sleep score and count; sleep interval scoring, missingness, axis X, Y and Z counts; VM; non-wear scoring according to majority algorithm, wear sensor, Choi, Troiano, van Hees algorithms; % of algorithms which indicated row was non-wear. Consensus was reached on the periods that were considered to be non-wear periods by the group.

Independent scorers then used this training session to score non-wear for their assigned participants. The true non-wear intervals generated by the quality control process were added to the 60 second epoch file, which was then used to generate the accuracy, positive predictive value, sensitivity and specificity metrics, as well as inter-rater reliability for files which were scored by two researchers.

### Combining Sleep and Non-wear

We combined sleep and non-wear at the epoch level, where each minute was scored for both sleep and non-wear, and at the interval level, where we calculated the number of minutes of missingness or non-wear according to the majority algorithm for each detected sleep interval, and percentage of minutes of missingness or non-wear according to the majority algorithm for each detected sleep interval.

**Addressing Data Missingness**

For instance, two R packages used to analyze actigraphy data-- the *actigraph.sleepr* package, which implements sleep scoring and detection algorithms, and the *nparACT* package, which computes non-parametric circadian activity rhythm analysis variables – require data to be complete at the time of analysis. In the context of actigraphy data, missingness can be thought of at two levels: at the epoch level, where individual epochs of data that are used to calculate aggregated variables (e.g., non-parametric circadian activity rhythm analysis variables) may be missing, and at the aggregated variable level, where aggregated variables for entire days/intervals may be missing.

If data were not available in the 3 days before and 3 days following the day with the missing data, we shifted the 7-day window accordingly (e.g., 2 days prior and 4 days following the day with missing data, if the 3 days prior day was not available for analysis).

This approach was applied to obtain interval and epoch-level sleep-wake scoring, and epoch-level non-wear scoring from the *actigraph.sleepr* package. Note that the imputed data were removed prior to running the non-wear statistics at the minute-by-minute level, and data were considered as missing.

**Advantages of using raw data and epoch-aggregated data**

The advantages of epoch-aggregated data include efficient storage and intuitive interpretability. In contrast, the raw accelerometer output is typically in units of gravity (g), and is more difficult to interpret due to its size (e.g., 30 samples per second in the case of our sampling method). Additionally, the processing of raw files is more computationally demanding. For example, the year’s worth of epoch-aggregated count data is 525 600 lines long with a file size of 67MB, but the raw gt3x file consists of nearly 956 million lines and takes up 15 GB of memory.

**Overlap of non-wear detection methods**

Figure S3a shows overlap of non-wear detected by different algorithms at the day level, while Figure S3b shows the overlap of non-wear detected by different algorithms at the participant level.

**Relationship between sleep and depressive symptoms**

Figure S4 illustrates the relationship between data missingness or non-wear with depressive symptoms

| **Figure S1: Participants in the Wellness Monitoring Study** |
| --- |
| **** |

| **Figure S2: Quality Control of Majority Non-Wear Algorithm** |
| --- |
| a) Example of Visualization for Quality Control of Majority Non-Wear Algorithm |
|        |
| b) Boxplot of the Performance of Majority Algorithm at the Participant Level Compared to Other Non-Wear Detection Methods   |

| Table S1: Linear Mixed Models Predicting Day-level Algorithm Statistic | | | | | | | | | | | | | |
| --- | --- | --- | --- | --- | --- | --- | --- | --- | --- | --- | --- | --- | --- |
| Comparisons |  | **Accuracy** | | | **Sensitivity** | | | **Specificity** | | | **Positive Predictive Value** | | |
| Algorithm 1 | **Algorithm 2** | **Difference** | **Test** | **P** | **Difference** | **Test** | **P** | **Difference** | **Test** | **P** | **Difference** | **Test** | **P** |
| Wear Sensor | **Majority (3)** | -0.105 | -48.107 | <0.0001 | -0.0160 | -5.2190 | <0.0001 | -0.0818 | -43.9570 | <0.0001 | -0.3444 | -62.7790 | <0.0001 |
| Wear Sensor | **Majority (4)** | -0.105 | -47.95 | <0.0001 | -0.0150 | -4.7180 | <0.0001 | -0.0829 | -44.5090 | <0.0001 | -0.3468 | -63.2110 | <0.0001 |
| Wear Sensor | **Choi** | -0.098 | -44.854 | <0.0001 | -0.0173 | -5.2190 | <0.0001 | -0.0730 | -39.2730 | <0.0001 | -0.2910 | -53.0350 | <0.0001 |
| Wear Sensor | **Troiano** | -0.077 | 35.327 | <0.0001 | -0.0380 | -12.1080 | <0.0001 | -0.0480 | -25.7220 | <0.0001 | -0.5264 | -9.5950 | <0.0001 |
| Wear Sensor | **Van Hees** | -0.103 | -47.116 | <0.0001 | 0.0160 | 4.9700 | <0.0001 | -0.0813 | -43.7070 | <0.0001 | -0.3319 | -60.4860 | <0.0001 |
| Majority (3) | **Majority (4)** | 0.0003 | 0.159 | 1 | 0.0016 | 0.5010 | 0.9962 | -0.0010 | -0.5520 | 0.9939 | -0.0024 | -0.4320 | 0.9981 |
| Majority (3) | **Choi** | 0.007 | 3.253 | 0.0145 | -0.00098 | -0.3120 | 0.9996 | 0.0087 | 4.6840 | <0.0001 | 0.0535 | 9.7440 | <0.0001 |
| Majority (3) | **Troiano** | 0.0279 | 12.779 | <0.0001 | -0.0220 | -6.8890 | <0.0001 | 0.3390 | 18.2350 | <0.0001 | 0.2918 | 53.1840 | <0.0001 |
| Majority (3) | **Van Hees** | 0.0005 | 0.159 | 1 | 0.0319 | 10.1870 | <0.0001 | 0.0005 | 0.2500 | 0.9990 | 0.0126 | 2.2930 | 0.1967 |
| Majority (4) | **Choi** | 0.007 | 3.094 | 0.0242 | -0.0025 | -0.8130 | 0.9652 | 0.0098 | 5.2360 | <0.0001 | 0.0558 | 10.1750 | <0.0001 |
| Majority (4) | **Troiano** | 0.0275 | 12.62 | <0.0001 | -0.0230 | -7.3900 | <0.0001 | 0.0350 | 18.7870 | <0.0001 | 0.2942 | 53.6160 | <0.0001 |
| Majority (4) | **Van Hees** | 0.0018 | 0.832 | 0.9617 | 0.0300 | 9.6860 | <0.0001 | 0.0015 | 0.8020 | 0.9672 | 0.0150 | 2.7250 | 0.07040 |
| Choi | **Troiano** | 0.021 | 9.527 | <0.0001 | -0.0210 | -6.5770 | <0.0001 | 0.0252 | 13.5510 | <0.0001 | 0.2383 | 43.4400 | <0.0001 |
| Choi | **Van Hees** | -0.0049 | -2.262 | 0.21 | 0.0330 | 10.5000 | <0.0001 | -0.0083 | -4.4340 | 0.0001 | -0.0409 | -7.4510 | <0.0001 |
| Troiano | **Van Hees** | 0.0257 | 11.79 | <0.0001 | 0.0530 | 17.0800 | <0.0001 | -0.0335 | -17.990 | <0.0001 | -0.2792 | -50.8910 | <0.0001 |

| **Table S2: Estimated Marginal Means for Linear Mixed Models of Algorithm Performance Statistics** | | | | | | | |
| --- | --- | --- | --- | --- | --- | --- | --- |
|  | **Wear Sensor**  Mean (SD) | **Wear Sensor**  Mean | **Choi Algorithm** Mean | **Troiano Algorithm** Mean | **van Hees Algorithm** Mean | **4-method Majority Algorithm: Wear Sensor, Choi, Troiano, Van Hees** Mean | **3-method Majority Algorithm: Wear Sensor, Choi, van Hees**  Mean |
| **Accuracy** | 0.886 | 0.984 | 0.963 | 0.989 | 0.991 | 0.991 | 0.991 |
| **Positive Predictive Value** | 0.631 | 0.922 | 0.683 | 0.963 | 0.978 | 0.975 | 0.975 |
| **Sensitivity** | 0.941 | 0.959 | 0.979 | 0.926 | 0.956 | 0.958 | 0.958 |
| **Specificity** | 0.917 | 0.990 | 0.965 | 0.998 | 1.00 | 0.999 | 0.999 |

| **Figure S3: Agreement between different methods of non-wear detection** |
| --- |
| 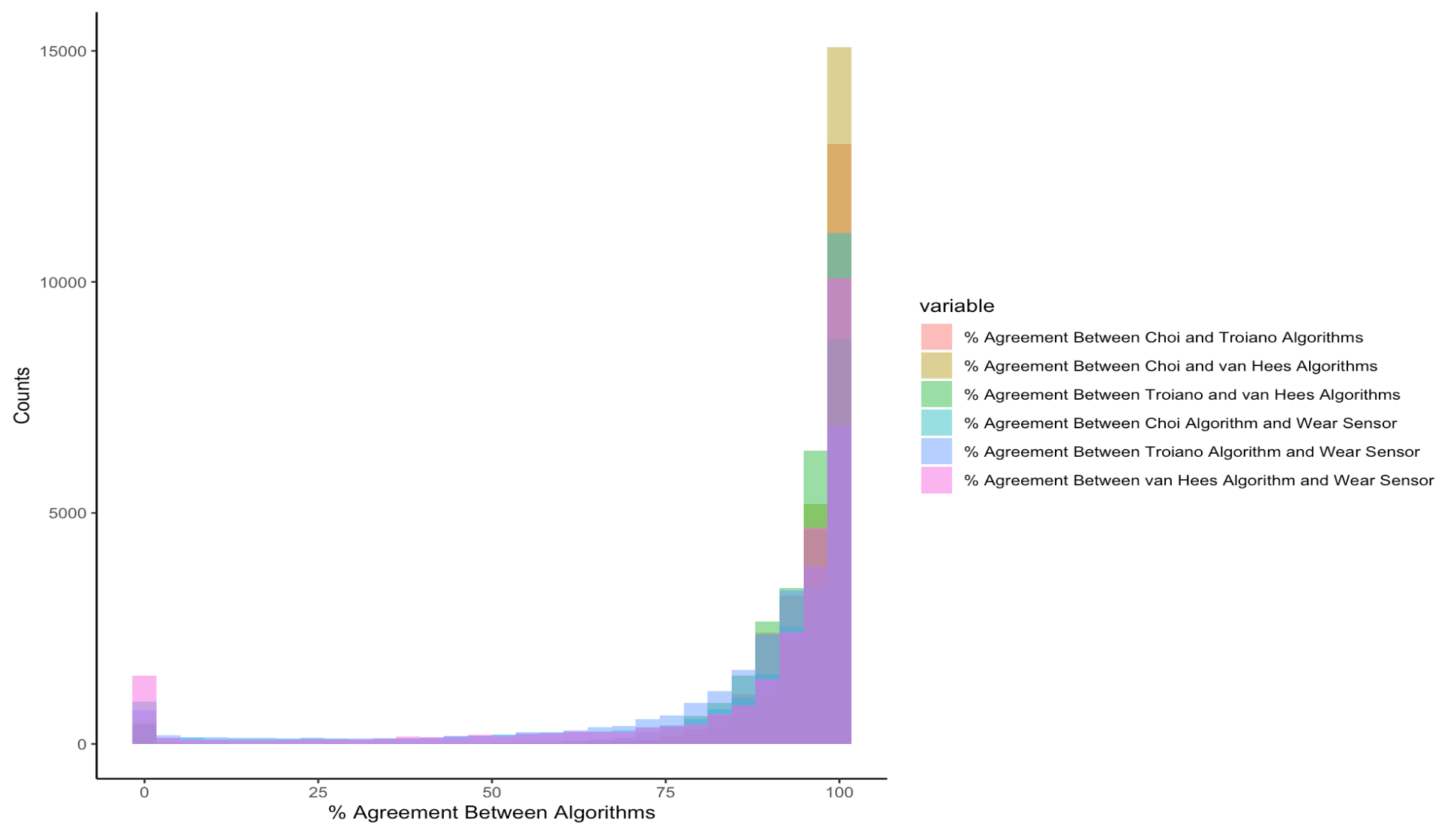   1. Count of days for which a certain percent agreement between individual methods of non-wear detection throughout the study was seen.   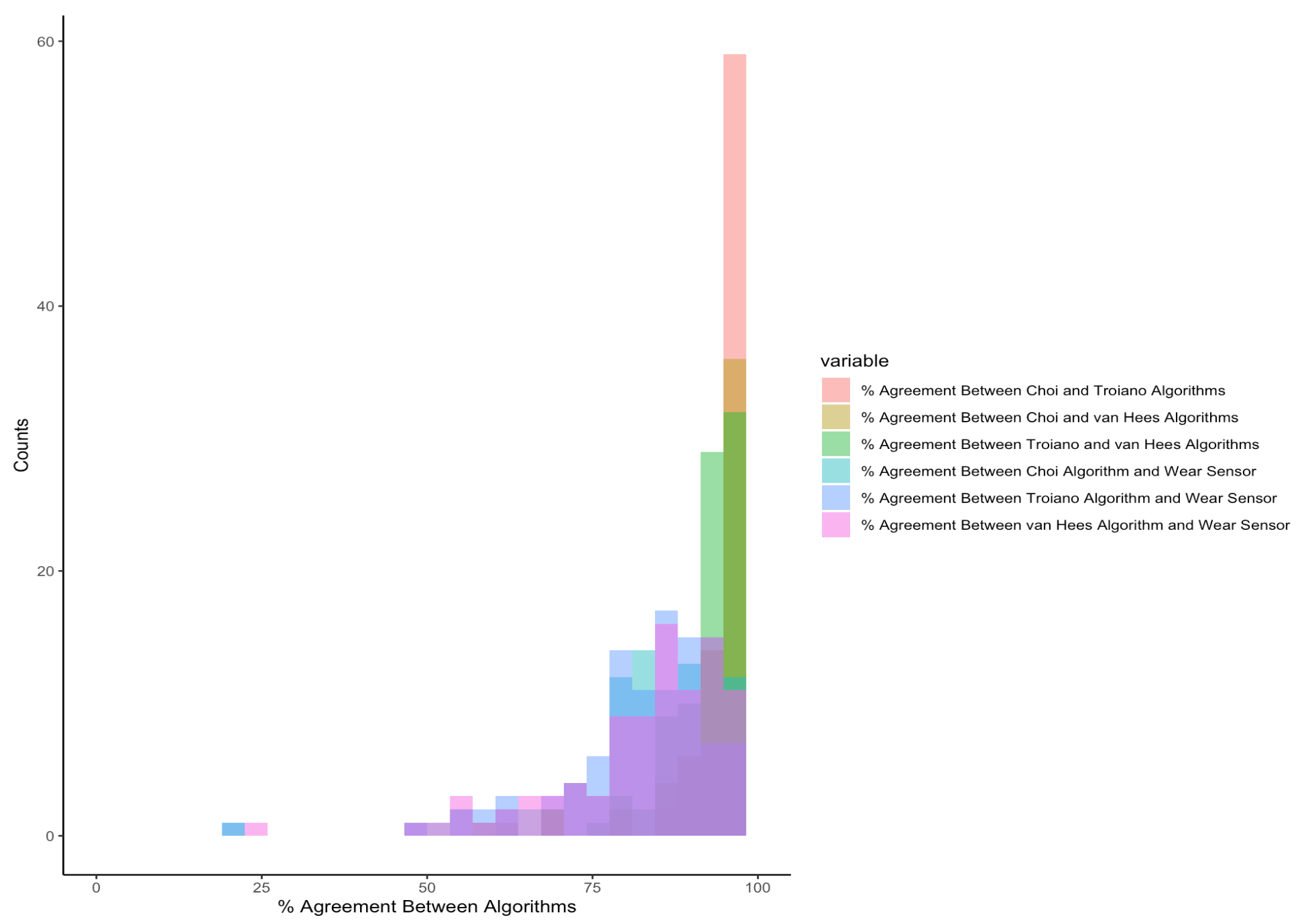   1. Percent agreement between individual methods of non-wear detection throughout the study summarized by participant. |
| 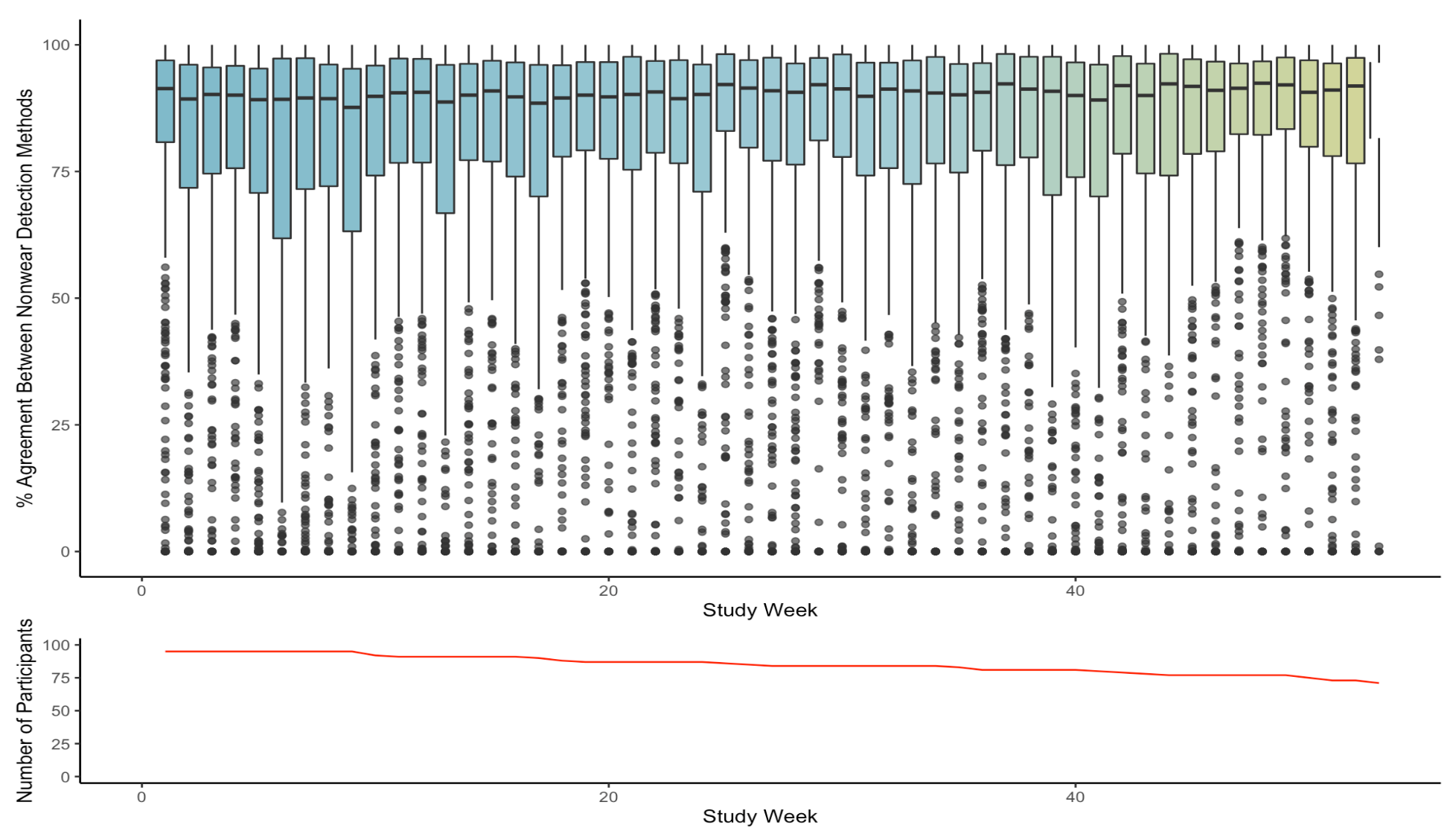 |
| 1. Percent agreement between methods of non-wear detection throughout study duration. There were no notable changes in agreement between methods of non-wear detection over time throughout study duration |

| **Figure S4: Relationship between Data Missingness or Non-wear and Depressive Symptoms** | |
| --- | --- |
|  | Average Per Day Per Period of Aggregation |
| 1. Missing | 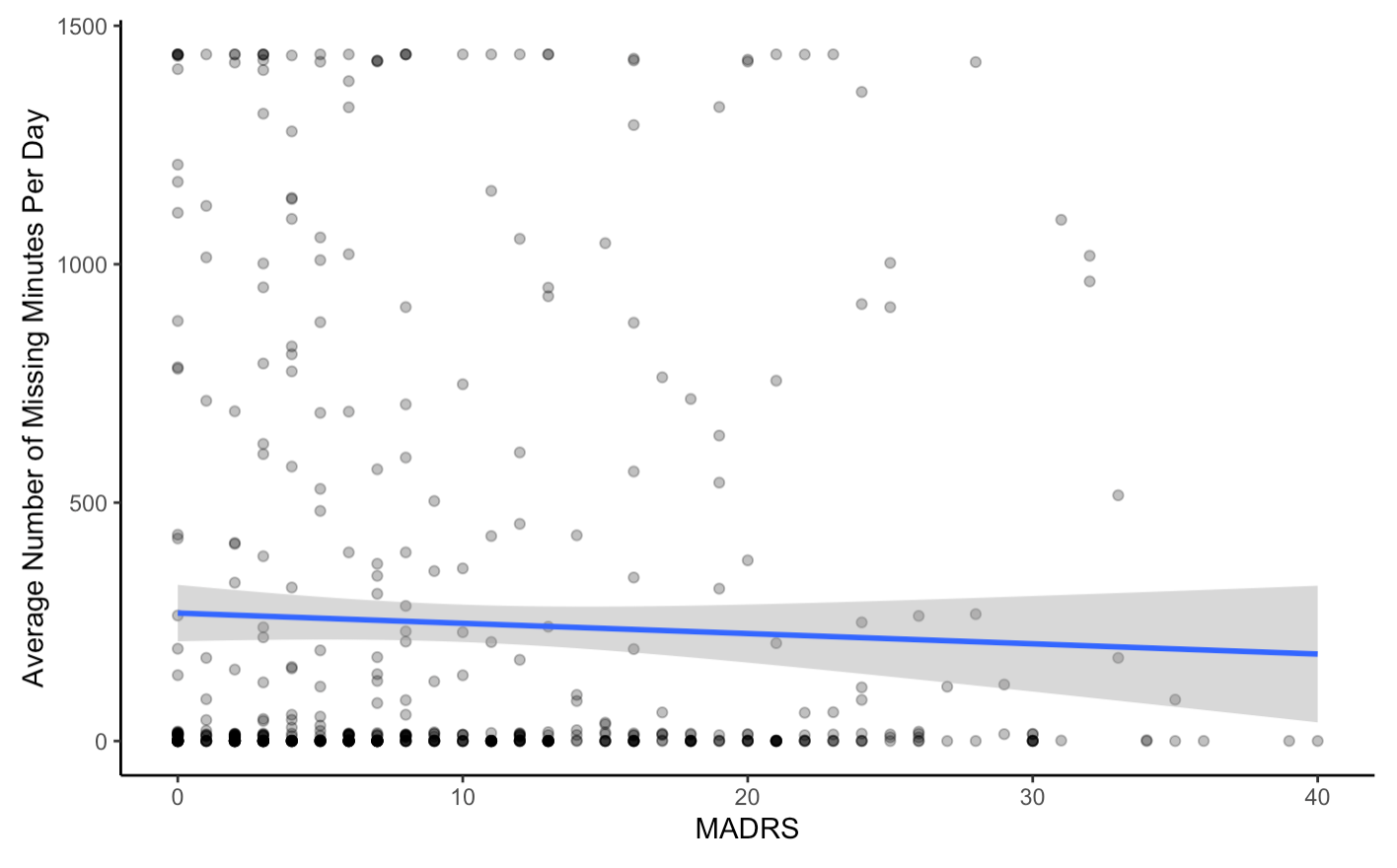  Rho=-0.04  P=0.36 |
| 1. Majority Algorithm | 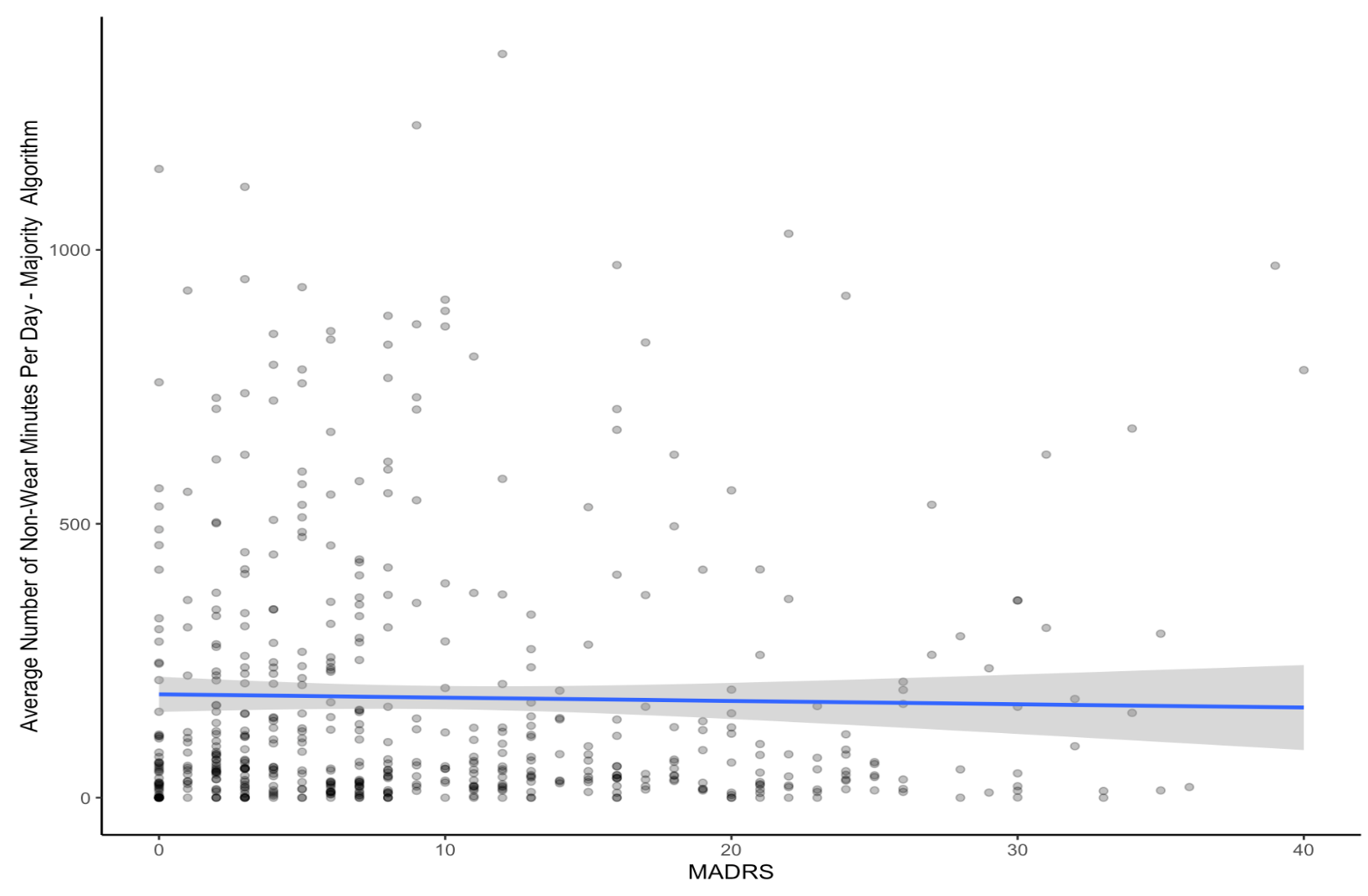  Rho=-0.02  P=0.63 |
| 1. Choi | 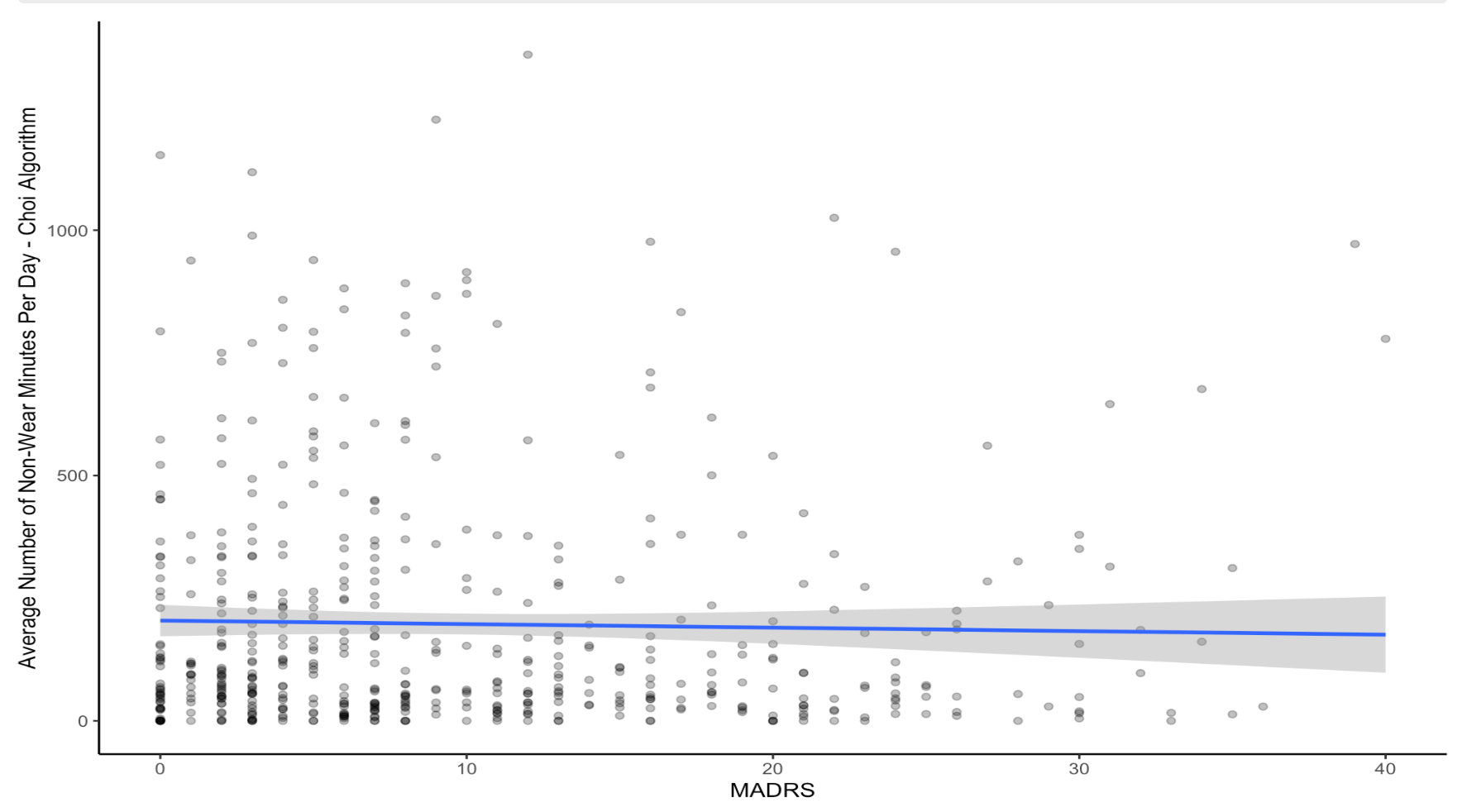  Rho=-0.03  P=0.57 |
| 1. Van Hees algorithm | 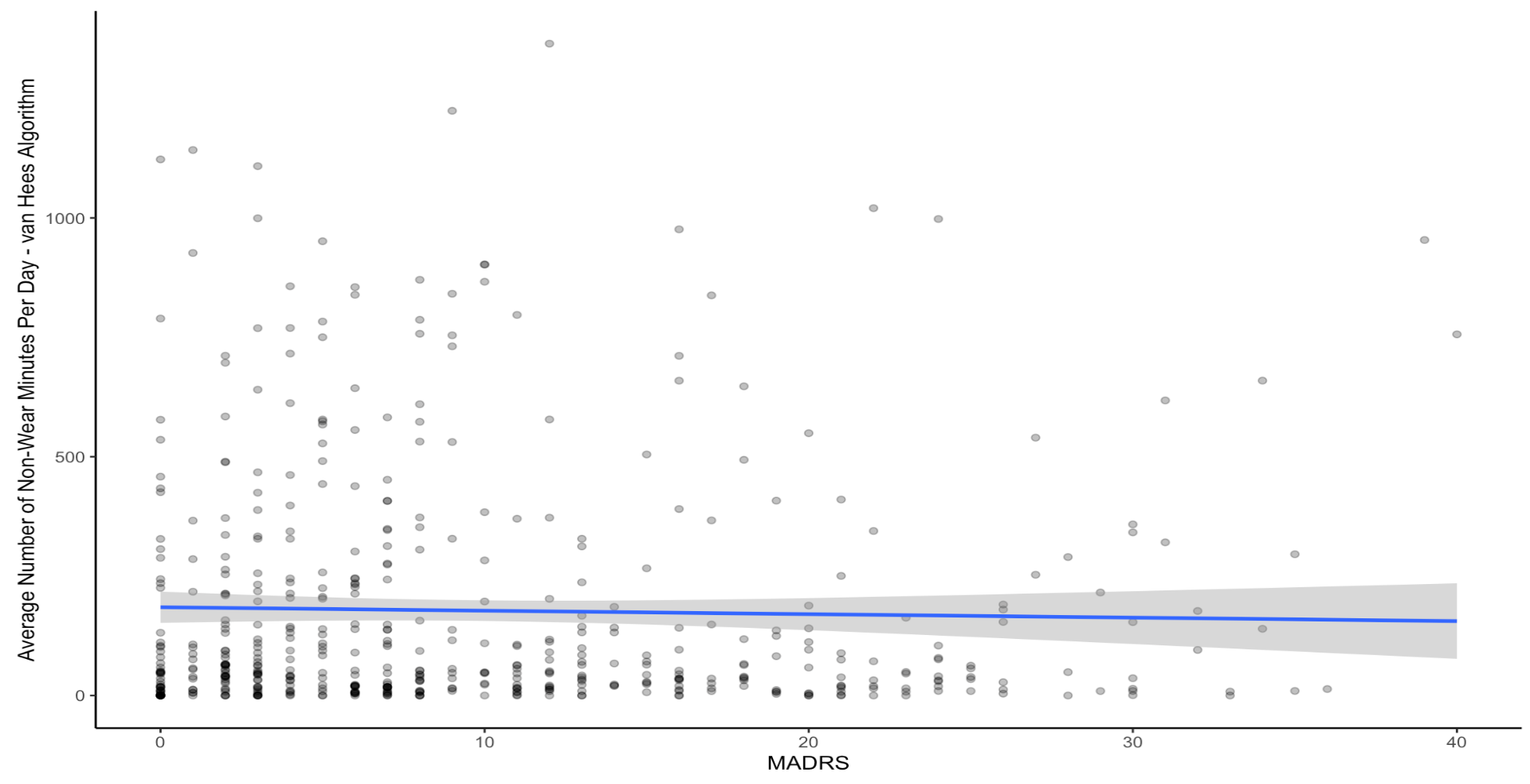  Rho= -0.02  P=0.57 |
| 1. Wear Sensor | **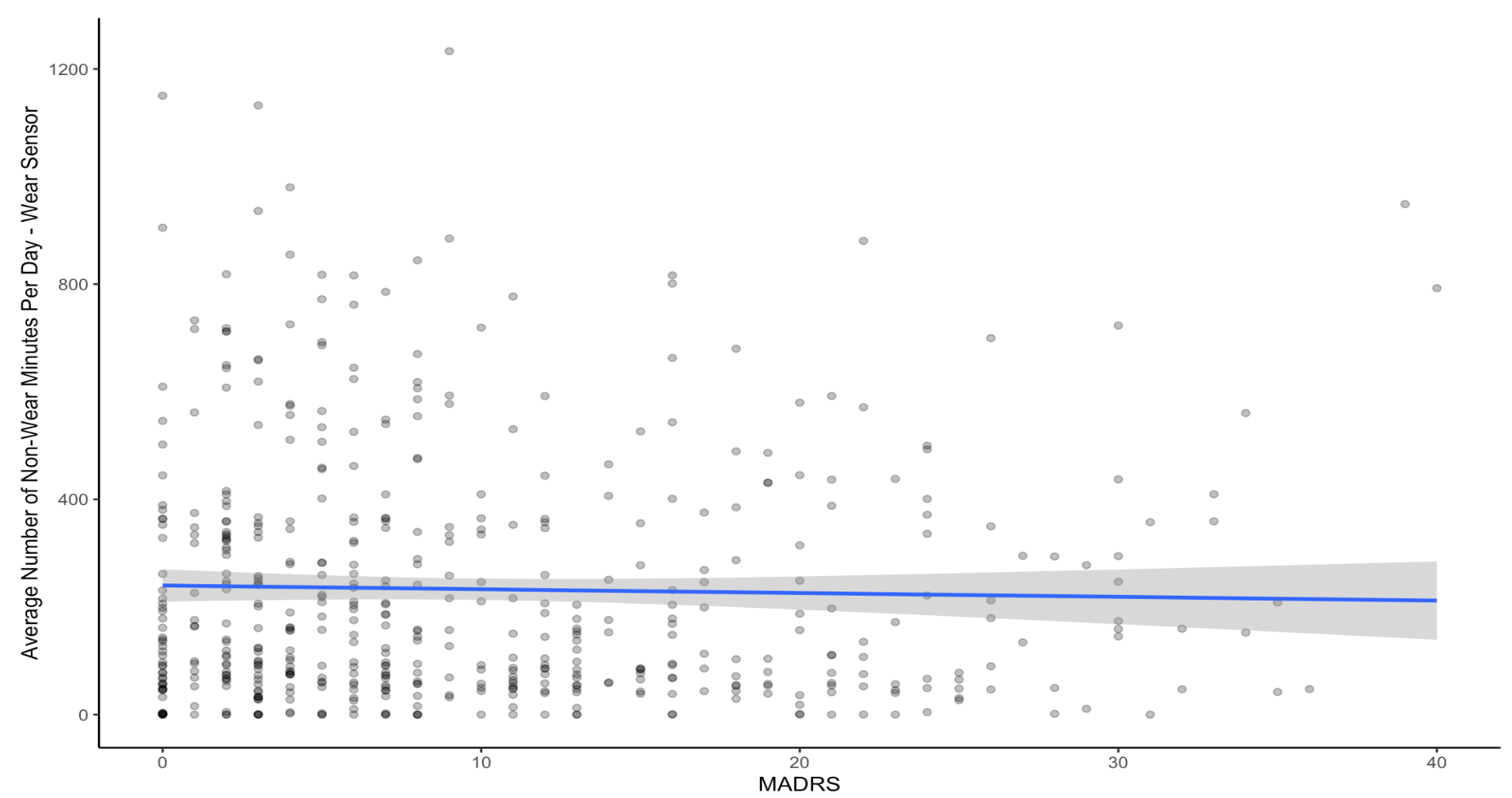**  Rho=-0.03  P=0.55 |

| **** |
| --- |
| **Figure S5: An illustration describing thresholding approach for excluding sleep intervals for the sensitivity analysis.** A total of 200 thresholds for excluding sleep intervals which overlapped with non-wear, and their combination with thresholds of number of hours of data per day for the day to be considered valid. We assessed whether these thresholds impacted the relationship of individual sleep metrics with depressive symptoms. Overlap thresholds were tested in 10% increments, ranging between <10% overlap and 100% overlap of sleep intervals with non-wear intervals. Valid day thresholds were tested in hourly increments ranging from >6 valid hours to 24 valid hours and all collected data, where a day had to have at least this number of hours of data available to be considered valid. Sleep intervals were detected by the Cole-Kripke and Tudor Locke algorithms. Wear/non-wear intervals were detected by 3-method majority algorithm. |
